# Supplementary material for: Mapping the evolution of fertility support policies in China: A content and instrumental analysis
Source: PLoS One. 2025 Oct 9;20(10):e0332137. doi: 10.1371/journal.pone.0332137 (PMC12510515; doi:10.1371/journal.pone.0332137)
Supplement: S1 Appendix — (ZIP) [file pone.0332137.s001.zip › S1 Appendix. 226 original policy documents/174-中华人民共和国未成年人保护法(FBM-CLI-1-5369).doc]

中华人民共和国未成年人保护法

**发布部门：** [全国人大常委会](https://www.pkulaw.com/law/chl?Aggs.IssueDepartment=201)

**发文字号：**中华人民共和国主席令第50号

**发布日期：**1991.09.04

**实施日期：**1992.01.01

**时效性：** [已被修改](https://www.pkulaw.com/law/chl?Aggs.TimelinessDic=03)

**效力级别：** [法律](https://www.pkulaw.com/law/chl?Aggs.EffectivenessDic=XA0101)

**法规类别：** [老少妇幼残保护](https://www.pkulaw.com/law/chl?Aggs.Category=01604)

**专题分类：** [未成年人保护](https://www.pkulaw.com/law/chl?Aggs.SpecialType=015)

**修改依据：**[中华人民共和国未成年人保护法(2006修订)](javascript:TMP(82812,0,'chl'))

[2012-2020对照](https://www.pkulaw.com/history/compare/对照-3500.html) [2006-2012编注](https://www.pkulaw.com/history/compare/编注-169.html) [1991-2006对照](https://www.pkulaw.com/history/compare/对照-168.html)

中华人民共和国主席令
（第五十号）

　　《中华人民共和国未成年人保护法》已由中华人民共和国第七届全国人民代表大会常务委员会第二十一次会议于1991年9月4日通过，现予公布，自1992年1月1日起施行。

中华人民共和国主席　杨尚昆
1991年9月4日

中华人民共和国未成年人保护法
（１９９１年９月４日第七届全国人民代表大会
常务委员会第二十一次会议通过）

第一章　总则

　　**第一条**　为了保护未成年人的身心健康，保障未成年人的合法权益，促进未成年人在品德、智力、体质等方面全面发展，把他们培养成为有理想、有道德、有文化、有纪律的社会主义事业接班人，根据[宪法](https://www.pkulaw.com/chl/5c498812eb87061ebdfb.html#class=alink)，制定本法。

　　**第二条**　本法所称未成年人是指未满十八周岁的公民。

　　**第三条**　国家、社会、学校和家庭对未成年人进行理想教育、道德教育、文化教育、纪律和法制教育，进行爱国主义、集体主义和国际主义、共产主义的教育，提倡爱祖国、爱人民、爱劳动、爱科学、爱社会主义的公德，反对资本主义的、封建主义的和其他的腐朽思想的侵蚀。

　　**第四条**　保护未成年人的工作，应当遵循下列原则：

　　（一）保障未成年人的合法权益；

　　（二）尊重未成年人的人格尊严；

　　（三）适应未成年人身心发展的特点；

　　（四）教育与保护相结合。

　　**第五条**　国家保障未成年人的人身、财产和其他合法权益不受侵犯。

　　保护未成年人，是国家机关、武装力量、政党、社会团体、企业事业组织、城乡基层群众性自治组织、未成年人的监护人和其他成年公民的共同责任。

　　对侵犯未成年人合法权益的行为，任何组织和个人都有权予以劝阻、制止或者向有关部门提出检举或者控告。

　　国家、社会、学校和家庭应当教育和帮助未成年人运用法律手段，维护自己的合法权益。

　　**第六条**　中央和地方各级国家机关应当在各自的职责范围内做好未年成人保护工作。

　　国务院和省、自治区、直辖市的人民政府根据需要，采取组织措施，协调有关部门做好未成年人保护工作。

　　共产主义青年团、妇女联合会、工会、青年联合会、学生联合会、少年先锋队及其他有关的社会团体，协助各级人民政府做好未成年人保护工作，维护未成年人的合法权益。

　　**第七条**　各级人民政府和有关部门对保护未成年人有显著成绩的组织和个人，给予奖励。

第二章　家庭保护

　　**第八条**　父母或者其他监护人应当依法履行对未年成人的监护职责和抚养义务，不得虐待、遗弃未成年人；不得歧视女性未成年人或者有残疾的未成年人；禁止溺婴、弃婴。

　　**第九条**　父母或者其他监护人应当尊重未成年人接受教育的权利，必须使适龄未成年人按照规定接受义务教育，不得使在校接受义务教育的未成年人辍学。

　　**第十条**　父母或者其他监护人应当以健康的思想、品行和适当的方法教育未成年人，引导未成年人进行有益身心健康的活动，预防和制止未成年人吸烟、酗酒、流浪以及聚赌、吸毒、卖淫。

　　**第十一条**　父母或者其他监护人不得允许或者迫使未成年人结婚，不得为未成年人订立婚约。

　　**第十二条**　父母或者其他监护人不履行监护职责或者侵害被监护的未成年人的合法权益的，应当依法承担责任。

　　父母或者其他监护人有前款所列行为，经教育不改的，人民法院可以根据有关人员或者有关单位的申请，撤销其监护人的资格；依照[民法通则](https://www.pkulaw.com/chl/4202520b3be0ae24bdfb.html#class=alink)第[十六条](https://www.pkulaw.com/chl/4202520b3be0ae24bdfb.html#%23tiao_16class=alink)的规定，另行确定监护人。

第三章　学校保护

　　**第十三条**　学校应当全面贯彻国家的教育方针，对未成年学生进行德育、智育、体育、美育、劳动教育以及社会生活指导和青春期教育。

　　学校应当关心、爱护学生；对品行有缺点、学习有困难的学生，应当耐心教育、帮助，不得歧视。

　　**第十四条**　学校应当尊重未成年学生的受教育权，不得随意开除未成年学生。

　　**第十五条**　学校、幼儿园的教职员应当尊重未成年人的人格尊严，不得对未成年学生和儿童实施体罚、变相体罚或者其他侮辱人格尊严的行为。

　　**第十六条**　学校不得使未成年学生在危及人身安全、健康的校舍和其他教育教学设施中活动。

　　任何组织和个人不得扰乱教学秩序，不得侵占、破坏学校的场地，房屋和设备。

　　**第十七条**　学校和幼儿园安排未成年学生和儿童参加集会，文化娱乐、社会实践等集体活动，应当有利于未成年人的健康成长，防止发生人身安全事故。

　　**第十八条**　按照国家有关规定送工读学校接受义务教育的未成年人，工读学校应当对其进行思想教育、文化教育、劳动技术教育和职业教育。

　　工读学校的教职员应当关心、爱护、尊重学生，不得歧视、厌弃。

　　**第十九条**　幼儿园应当做好保育、教育工作，促进幼儿在体质、智力，品德等方面和谐发展。

第四章　社会保护

　　**第二十条**　国家鼓励社会团体、企业事业组织和其他组织及公民，开展多种形式的有利于未成年人健康成长的社会活动。

　　**第二十一条**　各级人民政府应当创造条件，建立和改善适合未成年人文化生活需要的活动场所和设施。

　　**第二十二条**　博物馆、纪念馆、科技馆、文化馆、影剧院、体育场（馆）、动物园、公园等场所，应当对中小学生优惠开放。

　　**第二十三条**　营业性舞厅等不适宜未成年人活动的场所，有关主管部门和经营者应当采取措施，不得允许未成年人进入。

　　**第二十四条**　国家鼓励新闻、出版、广播、电影、电视、文艺等单位和作家、科学家、艺术家及其他公民，创作或者提供有益于未成年人健康成长的作品。出版专门以未成年人为对象的图书、报刊、音像制品等出版物，国家给予扶持。

　　**第二十五条**　严禁任何组织和个人向未成年人出售、出租或者以其他方式传播淫秽、暴力、凶杀、恐怖等毒害未成年人的图书、报刊、音像制品。

　　**第二十六条**　儿童食品、玩具、用具和游乐设施，不得有害于儿童的安全和健康。

　　**第二十七条**　任何人不得在中小学、幼儿园、托儿所的教室、寝室、活动室和其他未成年人集中活动的室内吸烟。

　　**第二十八条**　任何组织和个人不得招用未满十六周岁的未成年人，国家另有规定的除外。

　　任何组织和个人依照国家有关规定招收已满十六周岁未满十八周岁的未成年人的，应当在工种、劳动时间、劳动强度和保护措施等方面执行国家有关规定，不得安排其从事过重、有毒、有害的劳动或者危险作业。

　　**第二十九条**　对流浪乞讨或者离家出走的未成年人，民政部门或者其他有关部门应当负责交送其父母或者其他监护人；暂时无法查明其父母或者其他监护人的，由民政部门设立的儿童福利机构收容抚养。

　　**第三十条**　任何组织和个人不得披露未成年人的个人隐私。

　　**第三十一条**　对未成年人的信件，任何组织和个人不得隐匿、毁弃；除因追查犯罪的需要由公安机关或者人民检察院依照法律规定的程序进行检查，或者对无行为能力的未成年人的信件由其父母或者其他监护人代为开拆外，任何组织或者个人不得开拆。

　　**第三十二条**　卫生部门和学校应当为未成年人提供必要的卫生保健条件，做好预防疾病工作。

　　**第三十三条**　地方各级人民政府应当积极发展托幼事业，努力办好托儿所、幼儿园，鼓励和支持国家机关、社会团体、企业事业组织和其他社会力量兴办哺乳室、托儿所、幼儿园，提倡和支持举办家庭托儿所。

　　**第三十四条**　卫生部门应当对儿童实行预防接种证制度，积极防治儿童常见病、多发病，加强对传染病防治工作的监督管理和对托儿所、幼儿园卫生保健的业务指导。

　　**第三十五条**　各级人民政府和有关部门应当采取多种形式，培养和训练幼儿园、托儿所的保教人员，加强对他们的政治思想和业务教育。

　　**第三十六条**　国家依法保护未成年人的智力成果和荣誉权不受侵犯。

　　对有特殊天赋或者有突出成就的未成年人，国家、社会、家庭和学校应当为他们的健康发展创造有利条件。

　　**第三十七条**　未成年人已经受完规定年限的义务教育不再升学的，政府有关部门和社会团体、企业事业组织应当根据实际情况，对他们进行职业技术培训，为他们创造劳动就业条件。

第五章　司法保护

　　**第三十八条**　对违法犯罪的未成年人，实行教育、感化、挽救的方针，坚持教育为主、惩罚为辅的原则。

　　**第三十九条**　已满十四周岁的未成年人犯罪，因不满十六周岁不予刑事处罚的，责令其家长或者其他监护人加以管教；必要时，也可以由政府收容教养。

　　**第四十条**　公安机关、人民检察院、人民法院办理未成年人犯罪的案件，应当照顾未成年人的身心特点，并可以根据需要设立专门机构或者指定专人办理。

　　公安机关、人民检察院、人民法院和少年犯管教所，应当尊重违法犯罪的未成年人的人格尊严，保障他们的合法权益。

　　**第四十一条**　公安机关、人民检察院、人民法院对审前羁押的未成年人，应当与羁押的成年人分别看管。

　　对经人民法院判决服刑的未成年人，应当与服刑的成年人分别关押、管理。

　　**第四十二条**　十四周岁以上不满十六周岁的未成年人犯罪的案件，一律不公开审理。十六周岁以上不满十八周岁的未成年人犯罪的案件，一般也不公开审理。

　　对未成年人犯罪案件，在判决前，新闻报道、影视节目、公开出版物不得披露该未成年人的姓名、住所、照片及可能推断出该未年成人的资料。

　　**第四十三条**　家庭和学校及其他有关单位，应当配合违法犯罪未成年人所在的少年犯管教所等单位，共同做好违法犯罪未成年人的教育挽救工作。

　　**第四十四条**　人民检察院免予起诉、人民法院免除刑事处罚或者宣告缓刑以及被解除收容教养或者服刑期满释放的未成年人，复学、升学、就业不受歧视。

　　**第四十五条**　人民法院审理继承案件，应当依法保护未成年人的继承权。

　　人民法院审理离婚案件，离婚双方因抚养未成年子女发生争执，不能达成协议时，应当根据保障子女权益的原则和双方具体情况判决。

第六章　法律责任

　　**第四十六条**　未成年人的合法权益受到侵害的，被侵害人或者其监护人有权要求主管部门处理，或者依法向人民法院提起诉讼。

　　**第四十七条**　侵害未成年人的合法权益，对其造成财产损失或者其他损失、损害的，应当依法赔偿或者承担其他民事责任。

　　**第四十八条**　学校、幼儿园、托儿所的教职员对未成年学生和儿童实施体罚或者变相体罚，情节严重的，由其所在单位或者上级机关给予行政处分。

　　**第四十九条**　企业事业组织、个体工商户非法招用未满十六周岁的未成年人，由劳动部门责令改正，处以罚款；情节严重的，由工商行政管理部门吊销营业执照。

　　**第五十条**　营业性舞厅等不适宜未成年人活动的场所允许未成年人进入的，由有关主管部门责令改正，可以处以罚款。

　　**第五十一条**　向未成年人出售、出租或者以其他方式传播淫秽的图书、报刊、音像制品等出版物的，依法从重处罚。

　　**第五十二条**　侵犯未成年人的人身权利或者其他合法权利，构成犯罪的，依法追究刑事责任。

　　虐待未成年的家庭成员，情节恶劣的，依照[刑法](https://www.pkulaw.com/chl/29dd76714a5cc235bdfb.html#class=alink)第[一百八十二条](https://www.pkulaw.com/chl/29dd76714a5cc235bdfb.html#%23tiao_182class=alink)的规定追究刑事责任。

　　司法工作人员违反监管法规，对被监管的未成年人实行体罚虐待的，依照[刑法](https://www.pkulaw.com/chl/29dd76714a5cc235bdfb.html#class=alink)第[一百八十九条](https://www.pkulaw.com/chl/29dd76714a5cc235bdfb.html#%23tiao_189class=alink)的规定追究刑事责任。

　　对未成年人负有抚养义务而拒绝抚养，情节恶劣的，依照[刑法](https://www.pkulaw.com/chl/29dd76714a5cc235bdfb.html#class=alink)第[一百八十三条](https://www.pkulaw.com/chl/29dd76714a5cc235bdfb.html#%23tiao_183class=alink)的规定追究刑事责任。

　　溺婴的，依照[刑法](https://www.pkulaw.com/chl/29dd76714a5cc235bdfb.html#class=alink)第[一百三十二条](https://www.pkulaw.com/chl/29dd76714a5cc235bdfb.html#%23tiao_132class=alink)的规定追究刑事责任。

　　明知校舍有倒塌的危险而不采取措施，致使校舍倒塌，造成伤亡的，依照[刑法](https://www.pkulaw.com/chl/29dd76714a5cc235bdfb.html#class=alink)第[一百八十七条](https://www.pkulaw.com/chl/29dd76714a5cc235bdfb.html#%23tiao_187class=alink)的规定追究刑事责任。

　　**第五十三条**　教唆未成年人违法犯罪的，依法从重处罚。

　　引诱、教唆或者强迫未成年人吸食、注射毒品或者卖淫的，依法从重处罚。

　　**第五十四条**　当事人对依照本法作出的行政处罚决定不服的，可以先向上一级行政机关或者有关法律、法规规定的行政机关申请复议，对复议决定不服的，再向人民法院提起诉讼；也可以直接向人民法院提起诉讼。有关法律、法规规定应当先向行政机关申请复议，对复议决定不服再向人民法院提起诉讼的，依照有关法律、法规的规定办理。

　　当事人对行政处罚决定在法定期限内不申请复议，也不向人民法院提起诉讼，又不履行的，作出处罚决定的机关可以申请人民法院强制执行，或者依法强制执行。

第七章　附则

　　**第五十五条**　国务院有关部门可以根据本法制定有关条例，报国务院批准施行。

　　省、自治区、直辖市的人民代表大会常务委员会可以根据本法制定实施办法。

　　**第五十六条**　本法自１９９２年１月１日起施行。

　 附： 　　[刑法](https://www.pkulaw.com/chl/29dd76714a5cc235bdfb.html#class=alink)有关条文

　　第二十六条　教唆他人犯罪的，应当按照他在共同犯罪中所起的作用处罚。教唆不满十八岁的人犯罪的，应当从重处罚。
　　如果被教唆的人没有犯被教唆的罪，对于教唆犯，可以从轻或者减轻处罚。
　　第一百三十二条　故意杀人的，处死刑、无期徒刑或者十年以上有期徒刑；情节较轻的，处三年以上十年以下有期徒刑。
　　第一百八十二条　虐待家庭成员，情节恶劣的，处二年以下有期徒刑、拘役或者管制。
　　犯前款罪，引起被害人重伤、死亡的，处二年以上七年以下有期徒刑。第一款罪，告诉的才处理。
　　第一百八十三条　对于年老、年幼、患病或者其他没有独立生活能力的人，负有扶养义务而拒绝扶养，情节恶劣的，处五年以下有期徒刑、拘役或者管制。
　　第一百八十七条　国家工作人员由于玩忽职守，致使公共财产、国家和人民利益遭受重大损失的，处五年以下有期徒刑或者拘役。
　　第一百八十九条　司法工作人员违反监管法规，对被监管人实行体罚虐待，情节严重的，处三年以下有期徒刑或者拘役；情节特别严重的，处三年以上十年以下有期徒刑。

© 北大法宝：（[www.pkulaw.com](https://www.pkulaw.com/)) 专业提供法律信息、法学知识和法律软件领域各类解决方案。北大法宝为您提供丰富的参考资料，正式引用法规条文时请与标准文本核对。
欢迎查看所有[产品和服务](https://www.pkulaw.com/helps/2.html)。法宝快讯：[如何快速找到您需要的检索结果？](https://www.pkulaw.com/helps/70.html)[法宝](https://www.pkulaw.com/helps/69.html)V6有何新特色？

扫描二维码阅读原文

**原文链接：**<https://www.pkulaw.com/chl/f676777bde217880bdfb.html>
